# Evaluation of career planning group counseling and its effectiveness for intern male nursing students

**DOI:** 10.1186/s12909-022-03981-9

**Published:** 2023-01-17

**Authors:** Wen-Yao Xie, Xiao-Li Yang, Yi-Min Cai, Wei Mo, Zhou-Min Shen, Yu-Hui Li, Bi-Fang Zhou, Yu-Lian Li

**Affiliations:** 1 Interventional Operating Room, Hunan Provincial People’s Hospital (the First Affiliated Hospital of Hunan Normal University), Changsha, 410005 China; 2 Department of Liver Surgery, Hunan Provincial People’s Hospital (the First Affiliated Hospital of Hunan Normal University), Changsha, 410005 China; 3 Department of Nursing, Hunan Provincial People’s Hospital (the First Affiliated Hospital of Hunan Normal University), No. 61, Jiefang West Road, Changsha, 410005 China; 4 Interventional Vascular Surgery, Hunan Provincial People’s Hospital (the First Affiliated Hospital of Hunan Normal University), Changsha, 410005 China

**Keywords:** Career planning, Clinical nursing teaching, Group guidance, Male nursing students, Vocational maturity

## Abstract

**Objective:**

To investigate the influence of group counselling on the career planning and career maturity of male nursing students.

**Method:**

Sixty male nursing students were randomly selected from a specific-level first-class hospital in Hunan Province from July to August 2020 by using the convenience sampling method and were subsequently divided into the control group and the experimental group using the random number table method. The control group received routine pre-job training, including aspects concerning the hospital profile, nurse etiquette, nursing core systems, professional ethics, nursing emergency treatment and career prospects and planning. In the experimental group, career planning group counselling was added after the regular pre-service training (once a week) with each session lasting 2 h for a total of six training sessions. At six weeks and three months after the intervention, the career status evaluation scale and the college students' career maturity scale were used to compare the career planning and career maturity status of the two groups of male nursing students.

**Results:**

After six weeks and three months of intervention, all the dimensions and total scores of both the career status evaluation scale and the career maturity scale in the experimental group were superior to those in the control group with statistically significant differences (all *P* < 0.05). The repeated measures of variance analysis indicated that the differences in the total score for career planning and the four dimensions in terms of intergroup effect, time effect and interaction effect between the two groups were statistically significant (*P* < 0.05). The intergroup effect, time effect and interaction effect of the total score for vocational maturity, career goal, career confidence, career value, career freedom and career reference of the two groups were statistically significant (*P* < 0.05), while the time effect of the relative dependency dimension was also statistically significant (*P* < 0.05).

**Conclusion:**

Group counselling can significantly improve the career planning and career maturity status of male nursing students and has a certain long-term effect.

**Supplementary Information:**

The online version contains supplementary material available at 10.1186/s12909-022-03981-9.

## Introduction

Male intern nursing students form part of the strength of future nursing workforces. With the development of diversified nursing services in recent years, male nurses are favoured by both hospitals and society due to the advantages of greater physical fitness, stress resistance and resilience, creating a large demand for male nurses in hospitals [1]. Based on their physical strength compared to women, male nurses have a physical advantage in terms of working in various departments (e.g. emergency rooms, operating rooms and intensive care units) to care for patients and perform manual labour, and being more capable of taking on heavy workloads and night shifts. In addition, male physical strength provides an advantage in dangerous situations.

The gender-based expectations of men have driven Chinese male nurses to enter more prestigious departments or positions and to seek out higher salaries. However, nursing is still largely viewed as a female profession, meaning male nurses need to deal with gender bias and discrimination from both patients and the public, which usually related to persistent gender specific stereotypes [2]. Multiple studies have focused on patient and female colleagues' attitudes towards male nurses and have found that gender discrimination remains relatively common in the nursing profession, with male nurses being more likely to experience discrimination, isolation and difficulties in the work environment and to exhibit dissatisfaction with the practice environment [3, 4]. The deviation between social reality and professional ideals, high workloads and social pressures, as well as traditional perceptions, mean fewer males take up nursing as a lifelong career. Although the recruitment of male nursing students has expanded in recent years, there has not been a significant breakthrough in the number of males who eventually become registered nurses. A high turnover intention rate of male nurses is more than 83.3% for male nurses and the attrition rate among is as high as 68.72% [5], which eventually leads to a still modest proportion of clinical male nurses.

“Career planning” refers to the time, direction, and plan of action an individual sets to achieve his or her career goals. It can help trainee nurse students effectively solve the difficulties in the process of professional growth when taking into account their situation and immediate constraints. Thus, it can help reduce turnover decision and employment anxiety, improve career maturity, and increase the sense of career identity [6]. An existing study assessed the experience of male nursing students in the nursing profession by conducting two focus group interviews with junior and senior staff members. The results indicated that career planners should more actively promote the provision of a convenient learning environment for male nursing students, and highlighted the history of male nursing students in the classroom to motivate the present male nursing students [7].

Group counselling is an activity conducted by counsellors with relevant experience and a professional foundation in a group context. This process aims to imitate and learn new behavioural styles and attitudes and develop well-adapted behaviours through learning and observation [8]. This approach has unique advantages in terms of intervention effects concerning issues, such as career identity, subjective career barriers and low self-efficacy in career decision-making [9]. It was found that structured group psychological counselling had a good effect in estranging students from interpersonal barriers, improving interpersonal skills and coping styles, alleviating anxiety and depression and increasing members' self-efficacy [10].

Currently, there exist few intervention studies on career planning for male nursing interns [11]. Accordingly, the present study aimed to investigate the influence of group counselling on the career planning and career maturity of male nursing students to adopt the group counselling approach for developing a career planning intervention programme for male nursing interns. Overall, the results were highly promising, the details of which are described below.

## Methods

### Participants

In this study, 60 interns were randomly selected from 163 male nursing students in a tertiary hospital in Hunan Province from July to August 2020 and were subsequently assigned to the control (*n* = 30) and observation (*n* = 30) groups, respectively, using the random number table method. The randomisation process was performed using statistical analysis system software. The random numbers and groups were sealed in opaque envelopes and kept by the director of the nursing teaching and research department, who also helped supervise the random assignment process. A single-blind method was adopted, with the individuals involved in the routine pre-employment training, career planning group counselling and the collection and analysis of the questionnaires unaware of the grouping.

The inclusion criteria for the present study included male nurses who had been completing internships in 2020 with a duration of < 1 week who were willing to participate in this study and who were currently working on the clinical front line and had no communication barriers with others. The exclusion criteria included trainees who were unable to guarantee sufficient participation time or who were unwilling to voluntarily participate in the study, as well as those with an internship duration of > 1 week.

### Sample size calculation

A convenience sampling method was used to collect data for the study population. The sample size was calculated based on the Raosoft sample size calculator [http://www.raosoft.com/samplesize.html (accessed November 16, 2021)]. with an error range of 5% and a confidence level of 95% [12]. The sample size calculated was at least 27 for each group.

### Methods

#### Observation group

##### Establishment of the group counselling team

The team members were nursing professional teachers who had been trained in the area of group coaching and had counselling-related qualifications. The group counselling team comprised a total of seven individuals, including one director of the nursing teaching and research department, one officer of the nursing teaching and research department and five experts operating in various nursing-related fields, including nursing teaching, specialist nursing, nursing management, nursing research and scientific research design.

The leader of the group counselling activity was the director of the nursing teaching and research department, who had a PhD qualification, was a professor of nursing and mainly engaged in nursing education and nursing management. The leader was mainly responsible for guiding the design and implementation of the group counselling programme. The group counselling activity was overseen by the officer of the nursing teaching and research department, who had a master’s degree, was a nurse practitioner and was mainly engaged in nursing education. This individual was mainly responsible for the programme design and the coordination and hosting of the activity. Among the five group counselling activity instructors, one was a male clinical nursing instructor who had a master’s degree, was a vice professor of nursing, was mainly engaged in nursing teaching, and whose duties included analysing the form and characteristics of employment and helping male nursing students gain a comprehensive understanding of themselves and improve their knowledge of the nursing profession. The second instructor was a specialised male nurse who had a bachelor's degree, was a chief nurse mainly engaged in clinical acute and critical care nursing, and who shared his career history and career planning to help male nursing students strengthen their career goals, improve their career satisfaction and establish correct career values. The third instructor was a male nurse manager who had a master’s degree, was a vice professor of nursing and was mainly engaged in nursing management, teaching male nursing students about career planning and sharing their career planning history to help male nursing students better understand the importance of career planning and the nursing profession from multiple perspectives. The fourth instructor was a male nursing postgraduate student studying in the field of nursing education; their role was to increase the awareness among male nursing students of the importance of academic upgrading and of making multi-directional choices in their career planning. The final instructor was a male doctor of medicine research professional and a PhD supervisor who was primarily engaged in nursing teaching, as well as cultivating an interest in scientific research and promoting male nursing students’ comprehensive quality improvement.

##### Counselling programme development

This study combined Donald E. Super’s career development theory, Krumboltz’s social learning theory and Frank Parsons’ trait and factor theory (which formed the theoretical basis of this study), and designed various group activities around the core aspects of career planning, based on group counselling formation, including career identity, career satisfaction, organisational commitment, and a willingness to remain in the workplace [13]. The group counselling programme was designed to include six modules, with each module developed around an activity theme and including four sessions, i.e. warm-up activities, themed activities, summary and sharing, and homework assignment. Details of the specific implementation plan are provided in Table 1.

**Table 1:** Career planning group counseling program

Event Time	Event theme	Activity Objectives	Event Flow
Week 1	First meeting and welcome	①To promote understanding among team members, establish good interpersonal relationships, and build trust between team members and counselors as well as among team members ② To enable team members to understand the nature of the team, become familiar with the team rules, establish groups, and develop a team atmosphere and team norms	①Warm-up activity (40 min): “Interview with the stars” ②Theme activity (60 min): Establishing a team. First, the leader and counselor introduce themselves, then the counselor introduces the nature, goal and content of the team, reads the team code, and signs the team contract with the team members. Finally, groups are established, with 10 members in each group and 3 groups in total. Members of the group elect their own leaders and establish group names and slogans, and finally shout out slogans and show the group members' understanding of the group slogans ③Summary and sharing (15 min): Members first share the feeling of this activity in the group, and finally the group representatives give a summary speech. The counselor summarizes the situation of this theme activity ④ Homework assignment (5 min): influencing factors that affect your career choices
Week 2	Strengthen awareness and lead to reflection	①Strengthen the awareness of “career” and “occupation” among the members ② To make the team members more aware of the career choices they face, to inspire exploration of their careers and to make them think about the factors that affect their career development, and then to guide them to understand career planning and its significance	①Warm-up activity (40 min): Occupational quiz fun ②Theme activity (60 min): “Career Crossroads”. Distribute the “Career Crossroads” cards to each member. Members share and discuss the main factors that influence career development. Finally, the counselor introduces personal factors as the main factors affecting career development ③Summary and sharing (15 min): Members first share their feelings and gains of this activity in the group, discuss the importance of personal factors in career development, and finally the group representative give a summary speech. The counselor summarizes the situation of this theme activity ④ Homework assignment (5 min): Prepare a 1–2 min introduction of yourself
Week 3	Show charm, show your true self	①To help members to recognize themselves and the character, abilities, and personality traits of others ②To Guide members in self-exploration, strengthening self-affirmation, and the impact on career planning	②Warm-up activity (40 min): You and I in the mirror ②Theme activity (60 min): simulate employment interview session, each member (candidate) prepare 1–2 min of self-introduction, after the self-introduction, the counselor (examiner) ask “talk about the advantages and disadvantages of your application for nursing positions” and ask the candidate to answer on the spot ③Summary and sharing (15 min): The members share their feelings and gains of this activity, discuss the importance of self-awareness and career planning, and finally the group representatives make a summary speech. The counselor summarizes the situation of this theme activity ④ Homework assignment (5 min): Fill out the “Great Value Auction Checklist”
Week 4	Building concepts and emotions	①To enable team members to understand professional values, establish correct professional values, and thus reposition themselves ②To understand the team members' views on the nursing profession, to arouse their professional emotions, to cultivate professional pride, and to improve their professional identity and professional satisfaction	①Warm-up activity (40 min): “Professional values auction” ②Theme activity (60 min): Firstly, organize to watch the video of Nightingale's life and the glorious deeds of Chinese Tibetan male nurse Basang Dengzhu, the winner of Nightingale Medal, and then the team members express their views on nursing profession, guide the team members to establish correct professional values, cultivate professional pride, improve professional identity and enhance professional satisfaction ③Summary and sharing (15 min): Members first share their feelings and gains of this activity in the group, discuss the importance of values, and finally the group representatives make a summary speech. The counselor summarizes the situation of this theme activity ④Homework assignment (5 min): Make a career planning
Week 5	Helping with selection and planning	①To re-help the team members to understand their profession, improve their professional confidence, and stimulate their work enthusiasm and hard work spirit ②To help group members clarify their future direction through career interviews ③Introduce to the team members about career planning by combining the employment situation and employment characteristics of the nursing profession, to raise the members' awareness of career planning and help them formulate a career plan suitable for themselves	①Warm-up activity (30 min): “My career planning” ②Theme activity (70 min): Male nurses in clinical specialties, male nursing managers, male nursing postgraduates and research professionals (male) are invited to participate in career interviews, mainly to help group members answer their doubts about career planning in order to assist them in formulating a career plan suitable for themselves ③Summary and sharing (15 min): The group members share their feelings and inspirations of this activity: whether they saw their own shortcomings or their own shining points by listening to others' planning; whether they had a clearer career direction; whether they would try hard to implement the plan in the future. Finally, the group representatives make a summary speech. The counselor summarizes the situation of this theme activity ④ Homework assignment (5 min): summarize the harvest of the activity
Week 6	Summarize and enjoy the harvest	①Assist team members to share the gains of self-growth in the process, and to give appreciation and expectation to others, and to look forward to future development and progress together ②Summarize the group counseling activity	①Warm-up activity: “True Confessions” (50 min) ②Theme activity (60 min): Each member writes down their most memorable activity theme by reviewing the first five units, first communicating in small groups and finally sharing in large groups ③Fill in the questionnaire and take a group photo (10 min)

##### Implementing the counselling programme

The counselling activity was based on a variety of games, with each activity incorporating a different game to introduce the activity theme prior to forming small groups. This enabled the group members to participate in the themed activities in an open, accepting and supportive group atmosphere. After each activity, the counsellor summarised the proceedings, and the group members shared their feelings about the activity. Concerning the training method, the observation group participated in a one-week pre-employment training session for clinical practice that was organized by the nursing department. The training mainly included an introduction to the hospital, nursing etiquette norms and the core nursing system, as well as professional ethics, nursing emergency management and career prospect and planning. Following the pre-employment training, the male nursing students took turns to work in each clinical department according to the internship syllabus plan. Additionally, the observation group conducted a six-week career planning group counselling programme alongside the internship. The counselling sessions were provided Fridays from 19:00 to 21:00, once a week for a total of six training sessions.

#### Control group

The control group also participated in the pre-employment training on clinical internship, and participated in the internship rotation in each clinical department following the completion of the training. The internship department and internship content were the same as those used for the observation group. To prevent any mutual influence between the two groups, a different rotation order was used for each group.

### Evaluation tools

#### General information questionnaire

This questionnaire was self-created and included age, professional volunteering, education level and home location.

#### Career status assessment scale

The Chinese version of the career status assessment scale was translated by Tong Tian and comprised 23 items covering four dimensions, including 8 items on career identity, 5 to career satisfaction, 9 to organisational commitment and 1 to intention to remain in the workplace [14]. Each item was scored using a 5-point Likert scale, with each answer assigned a score of l–5 (completely disagree, relatively disagree, uncertain, relatively agree, and completely agree, respectively), while four items were reverse scored, for a total of 23–115 points. A higher score indicated a higher evaluation of occupational status. The Cronbach's alpha coefficients of scale and dimension ranged from 0.72 to 0.86, while the retest reliability was 0.82; the apparent content validity was 0.83, and the internal consistency coefficient was 0.86 [14]. Accordingly, the scale was deemed valid for use among nursing staff.

#### Career maturity inventory

The career maturity inventory was revised by Zhiyong Zhang et al. [15] The corresponding questionnaire comprised 34 entries (with a total of 170 points) covering 6 dimensions, i.e. 8 items on career goals, 6 on career confidence, 4 on career freedom, 6 on career values, 4 on reliance on friends and family and 6 related to career reference. Each item was rated according to a 5-point Likert scale, with scores ranging from 1–5 corresponding to highly non-conforming, non-conforming, unsure, conforming, and highly conforming, respectively. The scale had a retest reliability of 0.771, split-half reliability of 0.896 and an internal consistency reliability coefficient of 0.869, indicating overall good validity [3].

### Data collection

The group counselling team members distributed 30 copies of the career planning status questionnaire and the career maturity questionnaire among the observation group and the control group prior to the intervention and the six-week and three-month evaluation points. Uniform guidance language was used before distributing the questionnaires; all questionnaires were distributed on the spot and were successfully collected. All of the returned questionnaires were valid. To ensure the accuracy of the data analysis, the data analyst and program operators were blind during this study.

### Effect sizes

Effect sizes, based on the pooled pre- and post-test standard deviation to obtain a more precise estimate, were calculated to assess the effectiveness of group counseling interventions. Odd ratio were converted to Cohen's d (as described by Morris [16, 17]).

$$\begin{array}{cc}d=\frac{{\overline{x} }_{1}-{\overline{x} }_{2}}{s}& s=\frac{\sqrt{\left({n}_{1}-1\right){s}_{1}^{2}+({n}_{2}-1){s}_{2}^{2}}}{{n}_{1}+{n}_{2}-2}\end{array}$$

In line with Cohen's classification, effect sizes were classified into five levels: trivial (Cohen's d ≤ 0.2), small (> 0.2), moderate (> 0.5), large (> 0.8), very large (> 1.3) [18].

### Statistical analysis

An Excel database was created, and the SPSS Statistics 21.0 software program was used for statistical analysis of the data. In terms of the quantitative variables, a normality (sphericity) test was performed; the normally distributed data were expressed as mean ± standard deviation (*x* ± s), while an independent samples t-test and a repeated measures analysis of variance (ANOVA) test were conducted to compare the differences between the groups (test level alpha = 0.05).

## Results

### Comparison of general information between the two groups

A total of 60 valid questionnaires were collected (recovery rate, 100%). The results indicated no statistically significant difference between the two groups in the comparison of general information, which included the age, professional volunteering experience, education history and home location of the male nursing students (*P* > 0.05). The baseline information of the two groups of male nursing students was both balanced and comparable (see Table 2).

**Table 2:** Comparison of general information between the two groups

Group	Number of people	Age	Only child		The first volunteer to apply		Professional volunteer			School attended		Serving as class officers		Home location	
			Yes	No	Yes	No	Voluntary Application	Dispensing	collect	Bachelor's degree	Specialized degree	Yes	No	Rural	Urban
Control group	30	20.33 ± 1.40	14	16	23	7	28	1	1	2	28	16	14	19	11
Observation group	30	20.03 ± 1.43	10	20	20	10	28	1	1	5	25	15	15	17	13
^2^/*t*		0.823	1.111		0.739		0.000			0.647		0.067		0.278	
P		0.414	0.292		0.390		1.000			0.421		0.796		0.598	

### Comparison of career planning status scores between the two groups

There was no statistically significant difference between the two groups in terms of total career planning score, career identity, career satisfaction, organisational commitment and willingness to remain in their professional role prior to the intervention (both values, *P* > 0.05) (Table 3).

**Table 3:** Comparison of career planning status scores between the two groups (score, $\overline{\mathrm x}\pm\mathrm S$)

Projects	Group	Pre-intervention	Pre-intervention *P*-value	Post-intervention		Time Effect		Between-group effects		Interaction Effect		Effect sizes (Cohen's d)
				After 6 weeks of intervention	After 3 months of intervention	*F*-value	*P*-value	*F*-value	*P*-value	*F*-value	*P*-value	
Total Score	Observation group	77.29 ± 5.09	0.238	92.00 ± 5.97	96.00 ± 6.51	23.437	< 0.001	20.414	0.001	29.674	< 0.001	0.89
	Control group	77.71 ± 5.50		77.57 ± 5.03	76.57 ± 5.26							
Professional Identity	Observation group	27.57 ± 2.76	0.579	30.43 ± 4.20	33.71 ± 4.27	5.166	0.023	6.982	0.021	10.041	0.002	0.56
	Control group	27.43 ± 1.99		27.29 ± 2.43	26.43 ± 1.40							
Career satisfaction	Observation group	18.71 ± 4.61	0.342	23.43 ± 1.90	24.71 ± 0.76	6.861	0.009	14.520	0.002	18.326	< 0.001	0.97
	Control group	18.57 ± 2.07		18.71 ± 2.69	16.29 ± 2.14							
Organizational Commitment	Observation group	30.57 ± 4.04	0.132	33.29 + 3.35	35.71 ± 2.87	4.775	0.023	14.399	0.003	22.764	< 0.001	1.32
	Control group	30.71 ± 3.35		27.86 ± 2.97	25.57 ± 2.76							
Willingness to stay at work	Observation group	3.14 ± 0.38	0.097	4.57 ± 0.53	5.00 ± 0.00	4.324	0.027	6.193	0.029	29.382	< 0.001	0.73
	Control group	3.00 ± 0.82		3.71 ± 1.11	2.86 ± 0.69							

The data on total career maturity, career identity, career satisfaction, organisational commitment and intention to remain in their professional role in both groups met the normal distribution and spherical test requirements (*P* > 0.05 for the spherical symmetry test). The repeated measures ANOVA results suggested that the time effect, the inter-group effect and the interaction between the inter-group effect and the time effect of total career maturity, career identity, career satisfaction, organisational commitment and intention to remain in their role were statistically significant (*P* < 0.05). Further analysis revealed that the career planning status of the observation group was better than that of the control group after six weeks and three months of intervention, and the difference was statistically significant (*P* < 0.05). After group counseling intervention, the Cohen's d scores of total career planning score, career identity, career satisfaction, organisational commitment and willingness of the participants were all > 0.5, indicating that the intervention has significant effectiveness in improving career planning status (see Table 3).

### Comparison of occupational maturity scores between the two groups

The total score for occupational maturity prior to the intervention was 106.60 ± 5.46 in the observation group and 106.80 ± 3.63 in the control group, and the difference was not statistically significant when comparing the two groups (*t* =  − 0.409, *P* = 0.693). The data on total career maturity, career goals, career self-confidence, career values, career freedom, family and friend dependence and career reference in both groups met the normal distribution and spherical test requirements (*P* > 0.05 for the spherical symmetry test). The repeated measures ANOVA results suggested that differences in the inter-group, time and interaction effects, respectively, between the total career maturity score and the five dimensions of career goals, career confidence, career values, career freedom and career reference were statistically significant (*P* < 0.05), with a statistically significant time effect for the dimension of family and friend dependence (*P* < 0.05). On comparing the occupational maturity of the two groups at different time points, it was found that the level of occupational maturity in the observation group was higher than in the control group after six weeks and three months of intervention, and the difference was statistically significant (*P* < 0.05). After group counseling intervention, the Cohen's d scores of total occupational maturity scores, career goals, professional confidence, career values, career freedom and career reference of the participants were all > 0.5, indicating that the intervention has significant effectiveness in improving occupational maturity (see Table 4).

**Table 4:** Comparison of occupational maturity scores between the two groups (score, $\overline{\mathrm x}\pm\mathrm S$)

Projects	Group	Pre-intervention	Post-intervention		Time Effect		between-group effects		Interaction Effect		Effect sizes (Cohen's d)
			After 6 weeks of intervention	After 3 months of intervention	*F*-value	*P*-value	*F*-value	*P*-value	*F*-value	*P*-value	
Total Score	Observation group	106.60 ± 5.46	116.80 ± 2.59	125.40 ± 1.67	14.686	0.002	16.621	0.004	20.178	0.001	0.79
	Control group	106.80 ± 3.63	108.60 ± 6.54	106.20 ± 5.02							
Career Goals	Observation group	24.20 ± 3.49	28.40 ± 2.19	34.00 ± 0.00	7.957	0.012	11.433	0.010	26.265	< 0.001	1.26
	Control group	24.40 ± 2.51	25.00 ± 1.73	24.40 ± 1.82							
Professional Confidence	Observation group	18.40 ± 3.58	12.20 ± 2.49	8.00 ± 1.58	14.250	0.002	15.906	0.004	12.611	0.004	1.14
	Control group	18.80 ± 1.92	19.20 ± 4.32	18.40 ± 3.13							
Career Values	Observation group	17.40 ± 4.10	25.20 ± 1.92	29.00 ± 1.41	24.085	< 0.001	28.343	0.001	11.849	0.004	0.94
	Control group	17.20 ± 3.90	16.60 ± 2.30	16.00 ± 3.67							
Career Freedom	Observation group	14.00 ± 1.87	17.60 ± 0.89	19.80 ± 0.45	9.262	0.005	20.084	0.002	18.123	< 0.001	0.57
	Control group	14.00 ± 2.00	13.60 ± 1.82	13.20 ± 1.92							
Reliance on friends and family	Observation group	11.60 ± 2.70	7.80 ± 2.28	5.60 ± 1.52	7.406	0.021	4.446	0.068	2.513	0.146	0.16
	Control group	12.00 ± 1.22	11.60 ± 1.67	11.20 ± 1.10							
Career Reference	Observation group	20.00 ± 2.83	25.60 ± 1.82	29.00 ± 1.00	21.154	0.001	6.748	0.032	10.161	0.011	0.85
	Control group	21.40 ± 2.41	22.60 ± 2.51	23.00 ± 1.87							

## Discussion

Due to the peculiarities of China's college entrance examination system, many male nurses are forced to transfer to nursing majors with lower requirements in medical schools, based on their unsatisfactory performance in the entrance examination [19]. While this mechanism of speciality assignment helps nursing schools to overcome the difficulty of enrolment to some extent [20], without appropriate guidance and role models, this guidance mechanism actually exacerbates the negative attitudes of male nursing students toward the nursing profession.

### Improving the career planning status of intern male nursing students through group counselling

Sun et al. [19] found that nursing interns experienced extreme work pressure during their first internship. When nursing interns work as nurses for the first time, the complexity of the nursing environment will give rise to high pressure; the various factors that can lead to difficulties in clinical practice will affect the quality of nurses’ clinical practice, thereby impacting the confidence of interns in the nursing profession [20–23].

Table 3 shows that there were significant time, group and interaction effects, respectively, for each dimension of the career planning status evaluation scale (*P* < 0.05). The total career scores and the scores for each dimension were significantly higher in the observation group than in the control group after six weeks and three months of intervention, and the difference was statistically significant (*P* < 0.05). The values of Cohen's d after the intervention were > 0.5, showing the potential of this group counseling intervention in behavior change. These results were similar to those obtained by Meiling Wan et al., suggesting that group counselling aimed at career planning could improve the career planning status of male intern nursing students [24]. This may have been related to the fact that, in the career planning group counselling programme for intern male nursing students, the team members engaged in a process that started with meeting and getting to know one another, to communicating, learning new knowledge and continuously deepening it, which improved the team members' enthusiasm and initiative for learning about career planning [25].

Among the learning modules, in the themed activity titled ‘Showing charm, showing your true self’, a mock job interview session allowed the nursing students to objectively evaluate themselves and observe the abilities of others. They also encouraged team members to learn from one another, complement each other's strengths and weaknesses, make progress together and strengthen their self-affirmation. In the ‘Strengthen awareness and lead to reflection’ module, the team members were guided to explore the impact of self-awareness on career development by observing the choices made by male nursing interns at a ‘career crossroads’ to discover factors that may affect their future career choices. In the activities of the ‘Building concepts and emotions’ and ‘Helping with selection and planning’ modules, the team members observed a biographical portrayal of Nightingale and the activities of Chinese Tibetan male nurses. This was aimed at inspiring the nursing trainees to identify with the nursing profession through these role models and to cultivate professional pride, improve professional satisfaction and stimulate their work enthusiasm, thereby helping to stabilise their frame of mind and engage in the nursing profession. This will, in turn, help them to gain a better understanding of their career and their future plans and support them to be more determined in their career goals.

### Improving the professional maturity of male nursing students through group counselling

‘Professional maturity’ refers to the degree to which individuals master the career development goals that are appropriate for their specific career development stage, including career decision-making knowledge and career decision-making attitudes [12]. Male nursing students will be exposed to a level of negative information during this stage of their clinical practice; they may find that their expectations do not match their actual lives and that they lack a sense of professional belonging. If these aspects are not adjusted and guided promptly, students’ level of professional maturity will be reduced, which will directly affect the future development of the field of clinical nursing as a whole [26].

As Table 4 shows, significant time, inter-group and interaction effects (*P* < 0.05, Cohen's d > 0.5) influenced the total scores for occupational maturity and the five dimensions of occupational goals, occupational confidence, occupational values, occupational freedom and occupational reference across both groups after six weeks and three months of intervention. The total scores for occupational maturity and the scores for each dimension in the observation group were significantly higher than those in the control group after six weeks and three months of intervention, and the differences were statistically significant (*P* < 0.05). These results were similar to those obtained by Hongbo Mao et al. [27] and indicated that career planning group counselling was beneficial for improving the career maturity of intern male nursing students.

In the career planning group counselling programme, the game ‘You and I in the mirror’ was used to help the nursing interns better understand themselves, clarify their interests, establish role models in their minds and set future career goals. Another game, ‘Values auction’, was aimed at gaining a better understanding of the professional values of male nursing interns and helping them to better understand the importance of professional values, as well as actively guiding them to establish the correct professional values to support them to make better career decisions.

By inviting experts and talents in the field of nursing to participate in the career interviews, the authors were able to help the male nursing interns gain clearer goals for their nursing careers, inspiring them to identify with the value of nursing as a career and strengthening their self-confidence within the nursing career environment. The male nursing interns included in this study showed a stronger willingness to consider personal criteria when developing their ‘My career planning’ strategy, rather than relying on the opinions of friends and family, thus increasing their career autonomy. Overall, based on the results of this study, specific career planning counselling for intern male nursing students could significantly improve their career status and career maturity.

### Limitations and recommendations for future research

The primary limitation of this study was the use of the convenience sampling method, which limited the sample size and may have led to the introduction of sampling bias. In addition, since the entire duration of the study was six weeks, the intervention time was comparatively short. It is recommended that the number of group counselling sessions be increased in future intervention studies to enhance the effect of counselling. Furthermore, the participants in this study were mainly junior college students; it is recommended that groups involving individuals studying at different academic levels, such as undergraduate and postgraduate students, be included in future studies to verify the training effect of group counselling on nursing students at different academic levels.

## Conclusion

The investigation of the career planning of male nursing interns in clinical practice can help to better understand their psychological frame of mind. As part of an effective coping measure, group career counselling should be provided to help male intern nurses cope with occupational stress and reduce the attendant negative impact. This will also enable male trainee nurses to turn stress into motivation, adapt to and become integrated within the work environment and develop excellent professional skills. This will, in turn, provide fundamental grounding for the smooth development of the field of nursing as a whole.

## Supplementary Information

**Additional file 1. **CONSORT 2010 Flow Diagram.

## Data Availability

All data generated or analyzed during this study are included in this article.
