# Supplementary material for: Evaluation of career planning group counseling and its effectiveness for intern male nursing students
Source: BMC Med Educ. 2023 Jan 17;23:34. doi: 10.1186/s12909-022-03981-9 (PMC9847019; doi:10.1186/s12909-022-03981-9)
Supplement: Supplementary file 1 — Additional file 1. CONSORT 2010 Flow Diagram. [file 12909_2022_3981_MOESM1_ESM.doc]

**
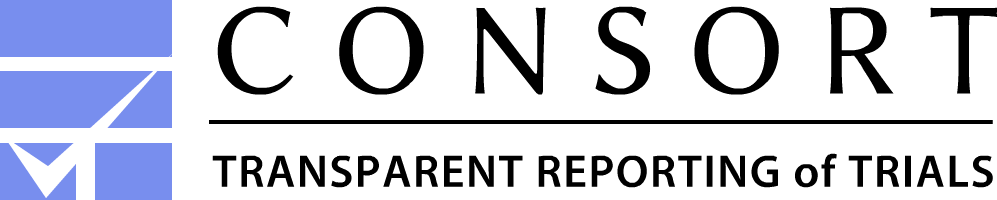
**

**CONSORT 2010 Flow Diagram**

**Allocation**

**Analysis**

**Follow-Up**

**Enrollment**

Assessed for eligibility (n = 73)

Excluded (n = 13)

♦  Not meeting inclusion criteria (n = 8)

♦  Declined to participate (n = 4)

♦  Other reasons (n = 1)

Analysed (n = 30)
♦ Excluded from analysis (give reasons) (n = 0)

Lost to follow-up (give reasons) (n = 0)

Discontinued intervention (give reasons) (n = 0)

Allocated to control group (n = 30)

♦ Received allocated intervention (n = 30)

♦ Did not receive allocated intervention (give reasons) (n = 0)

Lost to follow-up (give reasons) (n = 0)

Discontinued intervention (give reasons) (n = 0)

Allocated to observation group (n = 30)

♦ Received allocated intervention (n = 30)

♦ Did not receive allocated intervention (give reasons) (n = 0)

Analysed (n = 30)
♦ Excluded from analysis (give reasons) (n = 0)

Randomized (n = 60)
